# CRISPR/Cas9-based genome-wide screening for metastasis ability identifies FCGR1A regulating the metastatic process of ovarian cancer by targeting LSP1

**DOI:** 10.1007/s00432-024-05837-9

**Published:** 2024-06-15

**Authors:** Yingying Qi, Weiyan Zhu, Kexin Mo, Hui Jiang

**Affiliations:** 1 Department of Gynecology, The Fifth Affiliated Hospital of Guangzhou Medical University, Guangzhou, China; 2 Department of Obstetrics and Gynecology, The Sixth Affiliated Hospital of Guangzhou Medical University, QingYuan, China; 3 Department of Gynecology and Obstetrics, Guangdong Second Hospital of Traditional Chinese Medicine, Guangzhou, China

**Keywords:** CRISPR/Cas9 screening, Metastasis, FCGR1A, LSP1, Ovarian cancer

## Abstract

**Background:**

Metastasis is a main cause of death from ovarian cancer (OC). Identifying key markers involved in OC metastasis can aid in the effective detection of early postoperative metastasis. However, the role of FCGR1A in OC metastasis has yet to be fully established. A genome-wide CRISPR/Cas9-based screening system was used to identify regulatory factors involved in metastasis.

**Methods:**

The expression of FCGR1A and LSP1 in ovarian cancer cell lines was examined by quantitative real-time polymerase chain reaction (qRT‒PCR). The functions of FCGR1A and LSP1 in OC cell migration, invasion and proliferation were determined using wound healing, Transwell invasion and CKK-8 assays. A transcription-activated library was used to identify the potential downstream genes of FCGR1A. FCGR1A expression was detected by immunohistochemistry and the immunity risk score (IRS) scores were calculated.

**Results:**

FCGR1A was upregulated in OC cells compared with normal ovarian cells. Downregulation of FCGR1A inhibited metastasis, proliferation and epithelial–mesenchymal transition (EMT) progression in OC cells in vitro and intraperitoneal metastasis in vivo. Moreover, downregulation of FCGR1A was accompanied by decreased LSP1 expression. Overexpression of LSP1 partially reversed the tumor suppressive effect of FCGR1A downregulation. Higher FCGR1A expression was related to metastasis, higher grade, higher stage, and lymph node metastasis in OC. Survival analysis suggested that the group with higher FCGR1A expression had a lower tumor-free survival rate and a lower overall survival rate than did the group with low FCGR1A expression.

**Conclusions:**

FCGR1A enhances OC metastasis by regulating LSP1, and FCGR1A is associated with poor prognosis, suggesting that FCGR1A is a potential predictive factor for detecting early postoperative metastasis.

**Supplementary Information:**

The online version contains supplementary material available at 10.1007/s00432-024-05837-9.

## Introduction

Ovarian cancer (OC) ranks as the fourth most lethal tumor in women worldwide (Ronsini et al. 2023). In recent years, the application of targeted drugs has prolonged the survival of OC patients; however, although targeted drugs for OC mainly target BRCA mutations, the presence of BRCA1/2 or homozygous recombination-deficient somatic mutations accounts for only approximately 24% of OC patients (Pennington et al. 2013). The results of the SOLO1 study showed that the 7-year overall survival rate for patients receiving orapalib monotherapy for maintenance treatment was only 67% in patients with newly diagnosed OC with BRCA mutations, suggesting that the cotargeting of multiple genes may have more benefits for patients for whom single-gene targeting is ineffective (DiSilvestro et al. 2023). Therefore, although exploring the key genes that regulate metastasis is very important (Jia et al. 2021), no specific marker for detecting metastasis in early-stage OC is available.

Studies have shown that aberrant gene transcriptional regulation can lead to the suppression of macrophage function, which leads to immune escape, eventually resulting in metastasis or tumor progression (Jia et al. 2021). Transcriptional regulation is an important biological process that enables a cell or organism to respond to a variety of intracellular and extracellular signals and to coordinate cellular activities. These physiological processes are accomplished by interplay between numerous molecules in a network, including transcription factors, cofactors (coactivators and corepressors), and chromatin regulators, which are necessary for successful gene transcription (Casamassimi and Ciccodicola 2019). In the present study, we screened FCGR1A as a promoter of metastasis based on a genome-wide CRISPR/CAS9 library. Gene Ontology (GO) analysis revealed that FCGR1A is involved in transcriptional regulation. Analysis of the activation of this pathway suggested that FCGR1A is strongly correlated with EMT.

FCGR1A is a high-affinity receptor for fc-γ (the Fc segment of immunoglobulin G), which plays an important role in the immune response (Zhang et al. 2018; Bao et al. 2023). An increasing number of studies have shown that FCCR1A is associated with the development of numerous tumors, mainly through its involvement in regulating immune escape and metastasis, e.g., the excessive expression of FCCR1A in human and mouse induced pluripotent stem cell (IPSC)-derived endothelial cells (EESCs) helps tumor cells escape the cytotoxicity of antibody-dependent cells (ADCCs) (Scartozzi et al. 2010). An in vitro study showed that the bispecific antibody MDX-447, which targets FCGR1A, mediated the efficient lysis of breast cancer cells, and the results from phase II and phase III clinical trials of MDX-447 in prostate and breast cancers have shown some efficacy (Repp et al. 1995; Curnow 1997).

Lymphocyte-specific protein (LSP1) regulates macrophage phagocytosis and immune cell migration. LSP1 has recently been reported to be involved in the progression of many tumors. In melanoma, LSP1 decreases the migration and infiltration of T cells toward tumors, thus promoting tumor growth (Galon and Bruni 2019a). LSP1 also plays an important role in FCGR1A-mediated phagocytosis, and downregulation of LSP1 expression can severely reduce the phagocytic activity of macrophages (Maxeiner et al. 2015; Kaushal et al. 2023). However, there are few studies on the metastatic mechanism of FCGR1A and LSP in OC.

In this study, we screened the key gene FCCR1A, which promotes OC metastasis, based on a genome-wide CRISPR/CAS9 library. Furthermore, we verified the regulatory roles of FCCR1A and LSP1 in the metastasis, invasion and EMT progression of OC in vitro and in vivo. We propose that FCCR1A could serve as a promising target for the early identification of OC metastasis.

## Materials and methods

### Patient samples

OC tissue microarrays (TMRs, No. HOvaC151Su01) were purchased from Shanghai Zhuo Li Bio, China, and contained a total of 151 tissue samples from OC patients, including 32 patients with metastatic cancer, with information including the TNM stage, survival information, and clinical data.

## Cell culture

Human OC cell lines (A2780, SKOV3 and KGN) and a human normal ovarian epithelial cell line (IOSE80) were obtained from the American Type Culture Collection (ATCC, Manassas, VA, USA). The cells were cultured in RPMI-1640 medium supplemented with 10% FBS and CO_2_ at 37 °C.

## CRISPR screening

**Fig. 1:**
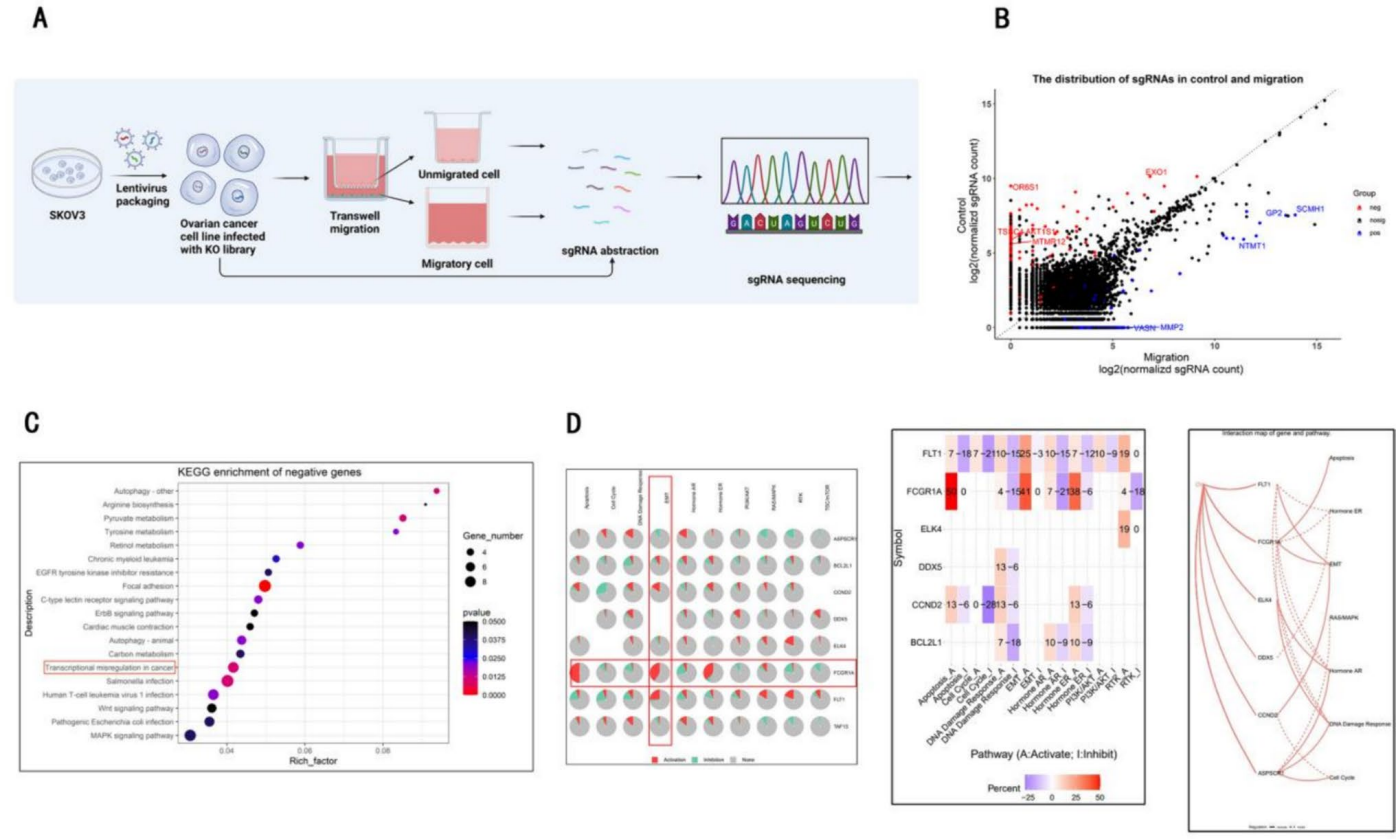
FCGR1A is involved in transcriptional misregulation and EMT progression based on the results of the bioinformatics analysis. **A** Flowchart for screening genes associated with OC metastasis based on the human GeCKO v2 CRISPR library. **B** The identified negatively and positively expressed genes (differentially expressed genes) based on Illumina sequencing. **C** KEGG pathway enrichment analysis revealed the top 20 pathways in which the DEGs were involved. **D** The pathway activity module revealed that FCGR1A was most strongly associated with the EMT pathway

In the genome-wide screen, SKOV3 cells were transduced with a pooled genome-wide lentiviral sgRNA library in a Cas9-containing vector (Addgene #1,000,000,100) at an MOI < 1. Stably transduced cells were selected with 1 μg/ml puromycin, and 220 million (M) cells were passaged every 72 h at a density of 5 M cells/15-cm dish for the duration of the screen. Then, we used a lentivirus-transfected SKOV3 cell line for the migration assay. We defined the cells that migrated to the lower chamber after 24 h as the group with greater metastasis, while the nonmigrating cells were defined as the group with weaker metastasis capacity. We collected cells from the upper and lower chambers separately and amplified them for the subsequent assay. The genomic DNA was harvested from 5 × 10^7^ cells, and the integrated sgRNA cassettes were amplified from the genomic DNA by PCR and subjected to massively parallel Illumina sequencing. Genes with a fold change in expression greater than 1 and a sgRNA count greater than 3 were chosen as potential genes related to the metastasis of OC cells (see Fig. 1A).

## Cell transfection

To knock down FCGR1A, three short hairpin RNA (shRNA) constructs, FCGR3A-sh-a (5′-GGTCATGAGAAGAAGGTAA-3′), FCGR3A-sh-b (5′-CCCTGAGTTGGAGCTTCAA-3′), and FCGR3A-sh-c (5′- CTCTGAATACCAAATACTA-3′), and a scrambled NC shRNA (5′-CTAAGGTTAAGTCGCCCTC-3′) were chemically synthesized at GeneChem (Shanghai, China). The shRNA sequences were synthesized, cloned and inserted into recombinant shRNA expression vectors using Lipofectamine 2000 (Invitrogen). Supernatants were collected after transfection for 72 h. Since the C locus had the greatest inhibitory effect, we ultimately selected FCGR3A-sh-c to knock down FCGR1A in OC cells for the subsequent experiments. To overexpress LSP1, mouse pLVSO5-LSP1 plasmids (Thermo Fisher Scientific), which were amplified using *Escherichia coli* DH5α and purified using the Plasmid Midi Kit (Qiagen), were transfected into the SKOV3 cell line using Lipofectamine 2000 (Life Sciences) according to the manufacturer’s protocol. Infected cells were selected by incubation with 4 μg/mL puromycin (Invitrogen) for 2 weeks to generate stably transfected cell lines.

## Wound healing assay

SKOV3 cells were seeded onto 6-well plates. After 24 h, the adhered cell monolayers were scratched with a 10-µL pipette tip and incubated in FBS-free medium for 24 h. The wound healing capacity was monitored via microscopy at 0 and 24 h. The wound widths in 5 random fields at the same timepoint were quantified using a microscope at × 40 magnification (MS60, Xinmei Optoelectronic Technology Co., Ltd., Guangzhou, China). The migration ability of the cells in each group was reflected by the mobility, which was calculated as follows: mobility = (0 h—other time points)/0 h.

## Invasion assay

SKOV-3 cells were plated in the uncoated wells of 24-well inserts for migration assays or in Matrigel-coated wells for invasion assays (BD Bioscience, Bedford, MA, USA). In total, 2 × 10^4^ cells/well were added to the upper chamber, and 500 μL of 10% FBS-containing medium was added to the lower chamber. Nonmigrating and migrated cells were collected for further study after 24 h of incubation, and the migrated cells in the lower chamber in 3 random fields were quantified using a microscope at × 200 magnification (MS60, Xinmei Optoelectronic Technology Co., Ltd., Guangzhou, China).

## Cell proliferation assay

Cell proliferation was analyzed by the Cell Counting Kit-8 (CCK-8, Beyotime, Shanghai, China) assay according to the manufacturer's protocols. Cells were seeded and cultured in 100 μL of medium in 96-well microplates at a density of 5 × 10^3^/well (Corning, USA). Ten microliters of CCK-8 reagent was added to each well, and the cells were then cultured for 2 h. The absorbance at 450 nm was analyzed using a microplate reader (Bio-Rad, Hercules, CA, USA). The experiments were repeated 3 times.

## Quantitative real-time polymerase chain reaction (qRT‒PCR)

Total RNA was extracted from OC cells using TRIzol reagent (Thermo Fisher Scientific, Inc.). cDNA was prepared using reverse transcriptase and an iScript™ cDNA Synthesis Kit (Bio-Rad Laboratories, Inc., Hercules, CA, USA) according to the manufacturer’s protocol. All PCR experiments were performed using SsoFast™ Eva Green Supermix (Bio-Rad Laboratories, Inc., California, USA) with the following thermal conditions: 95 °C for 30 s and 60 °C for 45 s for 45 cycles. The data were normalized to β-actin expression. Relative gene expression was calculated using the 2^−ΔΔ^CT method. The following primers were used for PCR: FCGR1A F: CACTGTTCTCTGGGTGACAATAC, R: TCTGTCTTCTTGAAGGCTGGAA; LSP1 F: AGGAGCACCAGAAATGTCAGC, R: GAGCGGTTTAGGGACTCGG; and β-actin-qF-ck F: CTACAGGGACGCCATCGAATC, R: AGCCCTCTTCAGCTTGTGTTG.

## Western blotting

Proteins were separated from cells by SDS‒PAGE. Then, the proteins were transferred to PVDF membranes. The membranes were incubated with primary antibodies for 3 h following the manufacturer’s instructions (E-cadherin 1:5000, HUABIO, China; N-cadherin 1:1000, Abclonal, China; Vimentin 1:20,000, HUABIO, China; or FCGR1A 1:500, ABclonal, China), followed by incubation with a secondary antibody for 1 h. GAPDH (ProteinTech, China) was used as the control. An enhanced chemiluminescence (ECL) protein blotting detection kit (human IgG) (Solarbio, China) was used for chemiluminescence detection.

## Strand-specific RNA-Seq library preparation and high-throughput RNA‑Seq

Total RNA extracted from the FCGR1A-sh SKOV3 cell line and the control SKOV3 cell line using TRIzol reagent (Invitrogen, Carlsbad, CA, USA). The RNA was treated with DNase I (DNA-free kit, Ambion, Texas, USA) twice at 37 °C for 30 min. Total RNA from each cell line was treated with reagents from the VAHTS Total RNA-Seq (H/M/R) Library Prep Kit for Illumina (Vazyme Biotech Co., Ltd., Nanjing, China) to remove the ribosomal RNA from each cell line. RNase R (Epicenter; 40 U) was used to purify the RNA at 37 °C for 3 h, after which the RNA was treated with TRIzol reagent. An RNA-Seq library was then constructed using the KAPA Stranded RNA-Seq Library Prep Kit (Illumina) and subjected to deep sequencing on an Illumina HiSeq 4000 instrument (Aksomics, Inc., Shanghai, China).

## Animal ethics statement and in vivo experiments

Four-week-old female BALB/c nude rats were purchased from Weitong Lihua Animal Technology Co., China (Shanghai, China) and sacrificed.

by an overdose of pentobarbital sodium. The animal license number for this experiment was SYXK 2018–0186. All animal experiments were approved by the Animal Care Committee of Guangzhou Medical University. The experiments were conducted in accordance with the Guide for Care and Use of Laboratory Animals (8th edition). An intraperitoneal xenograft mouse model was generated by transabdominally injecting SKOV-3 cells from different groups (0.2 mL/mouse, 3.0 × 10^7^ cells/ml). The weight and number of tumors were recorded 25 days after injection. The tissues from the model mice were stored for further studies.

## Immunochemistry (IHC)

The TMRs were immunohistochemically stained, and the immunity risk score (IRS) (Gorzelnik et al. 2023) was determined by multiplying the two scores below: ① the number of positive cells from five high-magnification fields chosen at random was calculated, and samples with a positive cell rate of 0%-10% scored 1, 10%-50% scored 2, 50%–75% scored 3, and 75%–100% scored 4; and ② the staining intensity was then scored as follows: samples with no positive staining were assigned a score of 0, those with light yellow or weak staining were assigned a score of 1, those with moderate staining intensity were assigned a score of 2, and those with strong staining intensity were assigned a score of 3.

## Statistical analysis

Statistical analysis and graphical presentation were performed using SPSS 26.0. All the results are presented as the means and standard deviations (means ± SDs) of at least three independent experiments. Comparisons between two groups were analyzed using Student’s *t* test. The results of three or more groups were analyzed by one-way analysis of variance (ANOVA).

## Results

### Screening of a genome‑wide pooled sgRNA library via transwell assay to detect potential genes involved in OC cell metastasis

To systematically identify potential genes associated with metastasis in OC, we performed a pooled genome-wide CRISPR/Cas9 (Addgene) knockout screen in the SKOV3 OC cell line. The human GeCKO v2 CRISPR library (Adelmann et al. 2019) contains 70,290 unique sgRNAs targeting 23,430 protein-coding genes and was used to generate a mutant OC cell pool by the transduction of SKOV3 cells at a low MOI (0.3), followed by puromycin selection. We defined the cells that migrated to the lower chamber after 24 h as the group with stronger metastatic capacity (Group 1), while the nonmigrating cells were defined as the group with weaker metastatic capacity (Group 2) (see Fig. 1A). After Illumina sequencing, we identified 314 negative genes (differentially expressed genes) as potential metastasis promoters (see attached Table 1).

**Table 1:** Correlation analysis between FCGR1A expression and clinicopathological features in ovarian cancer patients

Clinical features	TMA						
	Case	Low, n (%)	High, n (%)	*X*^2^	*P*	OR	95% CI
Tissue							
Primary tumor	135	100 (74.07)	35 (25.93)	12.651	< 0.001***	8.571	2.194–33.480
Metastatic tumor	12	3 (25.00)	9 (75.00)	–	–	–	–
Age (years)							
≤ 50	70	46 (65.71)	24 (34.29)	1.208	0.272	0.673	0.331–1.367
> 50	77	57 (74.03)	20 (25.97)	–	–	–	–
Pathological grade							
pT1–pT2	43	35 (81.40)	8 (18.60)	3.718	0.054	2.316	0.972–5.518
pT3–pT4	104	68 (65.38)	36 (34.62)	–	–	–	–
Tumor stage							
T1–T2	43	35 (81.40)	8 (18.60)	3.718	0.054	2.316	0.972–5.518
T3–T4	104	68 (65.38)	36 (34.62)	–	–	–	–
Lymph nodemetastasis							
N0	109	85 (77.98)	24 (22.02)	12.591	< 0.001***	3.935	1.801–8.599
N1	38	18 (47.37)	20 (52.63)	–	–	–	–
Distant metastasis							
M0	115	89 (77.39)	26 (22.61)	13.511	< 0.001***	4.401	1.931–10.030
M1	32	14 (43.75)	18 (56.25)	–	–	–	–

## FCGR1A is involved in transcriptional misregulation and EMT progression based on the results of bioinformatics analysis

Kyoto Encyclopedia of Genes and Genomes (KEGG) pathway analysis was performed on the DEGs. The top 20 ranked pathways are displayed Fig. 1B. The results indicated that the DEGs were mainly associated with autophagy, focal adhesion and transcriptional misregulation (Fig. 1C). Given the importance of transcriptional regulation in tumor immune escape and metastasis, we focused on this pathway and selected eight genes (ASPSCR1, CCND2, FLT1, ELK4, TAF15, DDX5, BCL2L1, and FCGR1A) for the subsequent experiments. Next, we used the pathway activity module (http://bioinfo.life.hust.edu.cn/web/GSCALite/) to explore the correlation of the above eight genes with hotspot pathways. Seven genes (ASPSCR1, CCND2, FLT1, ELK4, TAF15, BCL2L1, and FCGR1A) were associated with the EMT pathway. Among them, FCGR1A was most strongly associated with the EMT pathway (Fig. 1D).

## FCGR1A is critical for cell migration, invasion, proliferation and EMT progression in vitro

**Fig. 2:**
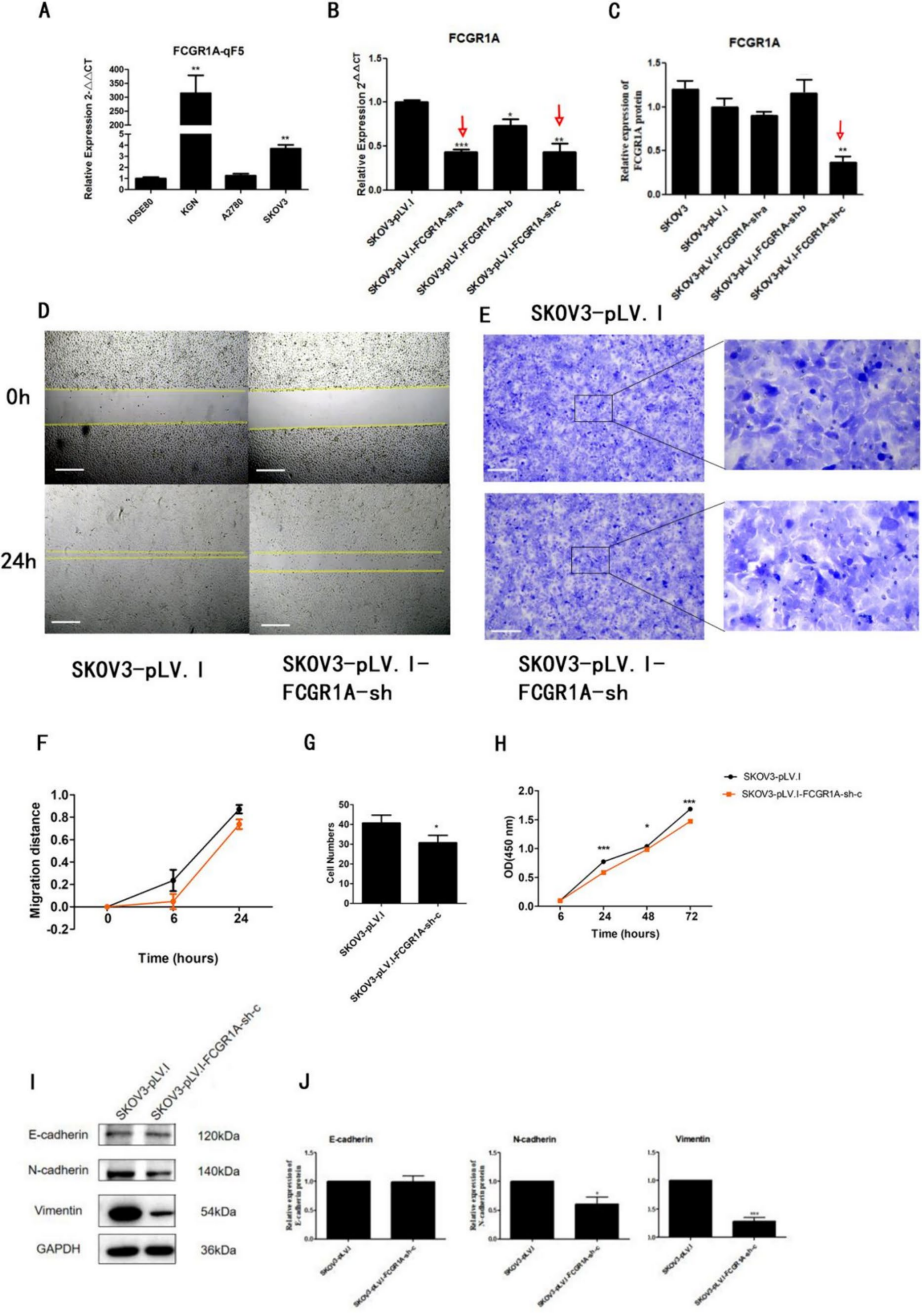
FCGR1A promotes the metastasis, invasion, and proliferation and EMT progression of OC cells in vitro. **A** qRT‒PCR analysis of FCGR1A in four cell lines. **B** and **C** qRT‒PCR and western blot analysis of FCGR1A in the indicated OC cell lines (n = 3 replicates). **D, E, F** and** G** Images and quantification of wound healing and Transwell invasion assays of shctrl and shFCGR1A SKOV3 cells (*n* = 3 replicates). (H) Quantification of CCK-8 assays of shctrl- and shFCGR1A-transfected SKOV3 cells (*n* = 3 replicates). **I** and **J** Images and quantification of western blot analysis of E-cadherin, N-cadherin and vimentin in shctrl and shFCGR1A SKOV3 cells (EMT, epithelial-mesenchymal transition. The error bars indicate the SD of the mean from three independent experiments, *p < 0.05, **p < 0.01 and ***p < 0.001 by unpaired, two-tailed student's *t*-test. Scale bar = 100 µm)

To determine whether FCGR1A affects cancer cell motility, we compared its expression levels in 3 OC cell lines (KGN, A2780, and SKOV3) and normal ovarian epithelial cells (IOSE80) and found that the expression of FCGR1A was greater in KGN and SKOV3 cells than in A2780 and IOSE80 cells (Fig. 2A). Since we carried out a CRISPR screen for potential metastasis-related genes in the SKOV3 cell line, we selected the SKOV3 cell line for use in the subsequent experiments.

The significant reduction in FCGR1A mRNA and protein expression indicated that the shRNA successfully induced the depletion of FCGR1A in the SKOV3 cell line (Fig. 2B, C).

To further investigate the role of FCGR1A in OC cell metastasis, we analyzed the motility and EMT in the FCGR1A knockdown and control groups. FCGR1A-depleted cells were then subjected to wound healing and Transwell invasion assays. The results from these assays suggested that the migration and invasion capacity of SKOV3 cells was significantly inhibited after FCGR1A gene knockdown (Fig. 2D–G). Next, we tested the effect of FCGR1A knockdown on proliferation by the CCK-8 assay, and the results revealed that knockdown of FCGR1A significantly reduced the growth of OC cells (see Fig. 2H). The WB results showed that the sh-FCGR1A group displayed reduced N-cadherin and vimentin expression compared with the control group, and the protein levels of N-cadherin and vimentin were markedly decreased (see Fig. 2I, J). In summary, FCGR1A may promote the metastasis, invasion, and proliferation of OC cells and EMT progression in OC.

## FCGR1A is critical for the abdominal metastasis of OC in vivo

**Fig. 3:**
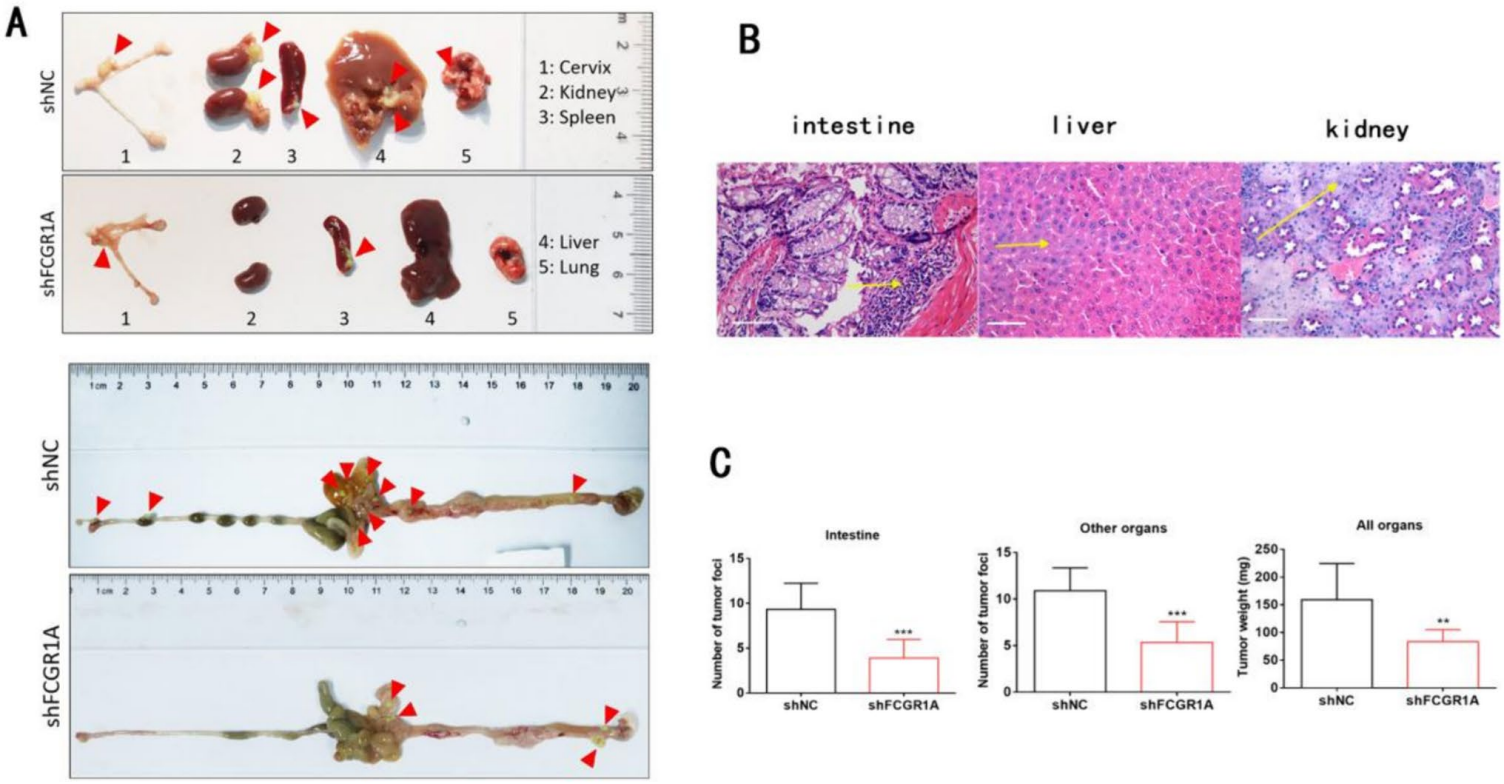
FCGR1A promoted abdominal metastasis of OC in vivo. **A** Xenograft models in nude mice treated with shctrl or shFCGR1A; the red arrow points to a metastatic focus. **B** H&E staining and quantification of liver and kidney metastatic lesions in mice inoculated with the indicated SKOV3 OC cells. **C** The numbers and weights of abdominal metastatic tumors in nude mice incubated with shctrl or shFCGR1A (n = 3 replicates. The error bars indicate the SD of the mean from three independent experiments, ***p < 0.001 by unpaired, two-tailed student's *t*-test. Scale bar = 100 µm)

To further confirm the role of FCGR1A in vivo, we established a xenograft model in nude mice using the SKOV3 cell line (Fig. 3A). The results suggested that an abdominal metastasis model was successfully constructed in both groups, as tumors distributed on the surface of the peritoneum, mesentery, and intestinal tubes were detected (see Fig. 3B). Then, the numbers and weights of the abdominal metastatic tumors were calculated. Downregulation of FCGR1A suppressed the intraperitoneal metastasis of OC cells, as indicated by the finding that mice inoculated with FCGR1A-sh SKOV3 cells developed fewer abdominal metastases than the control mice (9.22 ± 3.63 vs. 20.22 ± 4.71, *t* = 5.547, *p* < 0.001), and the total weight of the mice was significantly reduced (83.42 ± 21.65 vs. 159.13 ± 65.30, *t* = 3.301, *p* < 0.001) (see Fig. 3C).

## Transcriptomic sequencing analysis indicated that the expression of LSP1 was strongly correlated with the expression of FCGR1A

**Fig. 4:**
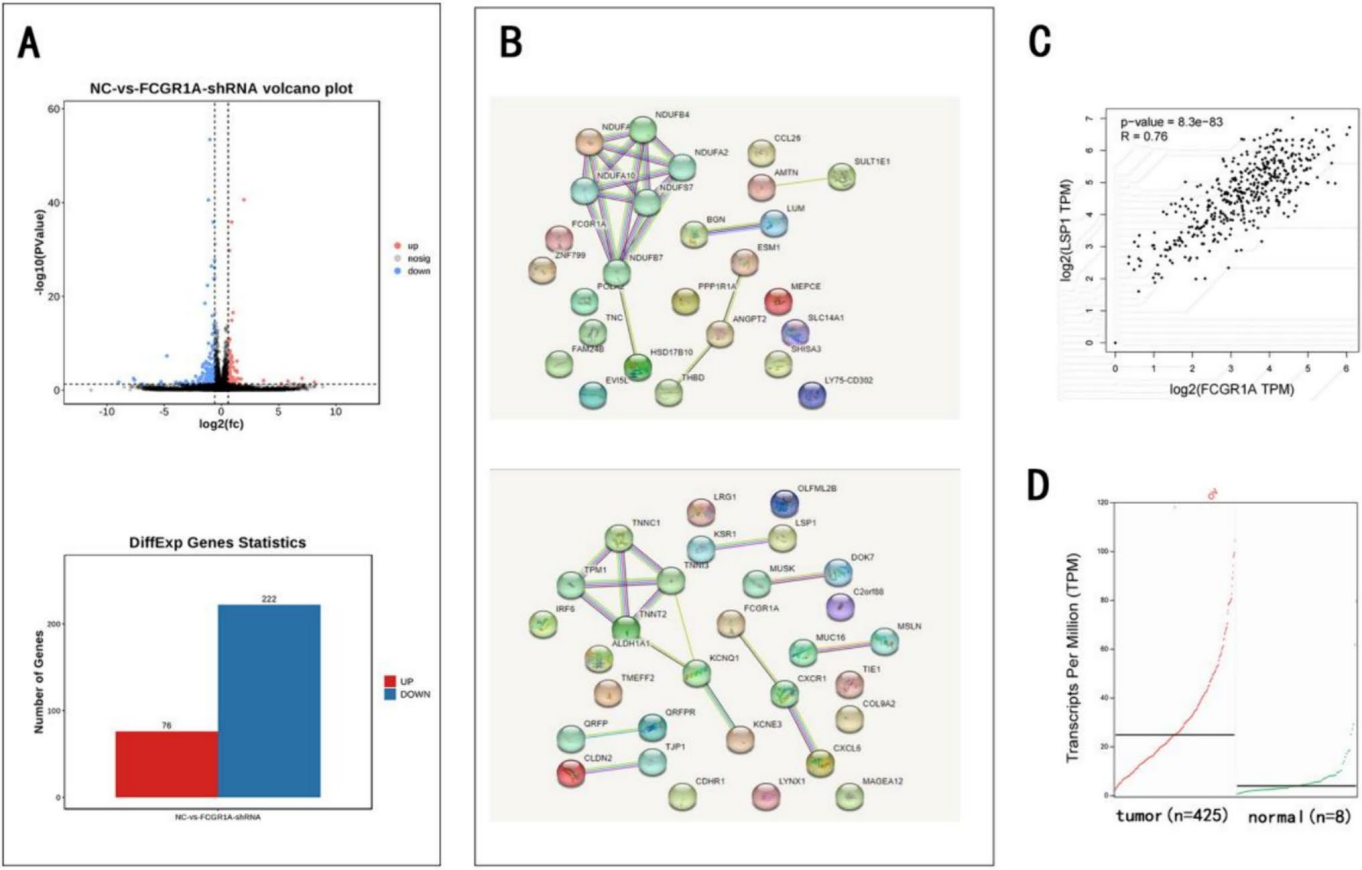
The results of the transcriptomic sequencing and TCGA database analyses indicated that the expression of LSP1 was strongly correlated with the expression of FCGR1A. **A** Transcriptome analysis of FCGR1A-silenced SKOV3 cells to screen the potential downstream genes regulated by FCGR1A. **B and C** LSP1 gene expression correlated with FCGR1A gene expression, as indicated by the Pearson correlation coefficient between LSP and FCGR1A gene expression. **D** Expression of the LSP1 gene in ovarian cancer ovaries compared with that in normal ovaries (*p < 0.05 by unpaired, two-tailed student's *t*-test)

To identify differentially expressed genes (DEGs) whose expression changes in response to fluctuations in FCGR1A gene expression, we performed an expression analysis of the FCGR1A-sh SKOV3 cell line and the control SKOV3 cell line using RNA-Seq. A total of 14,673 protein-coding genes were sequenced. Among these genes, 298 were deregulated, 61 were downregulated, and 222 were upregulated in the FCGR1A-sh-SKOV3 cell line compared to the control SKOV3 cell line (Fig. 4A).

The top 20 upregulated and downregulated genes were selected for further screening of potential downstream genes regulated by FCGR1A (see attached Table 2). Analysis of data from TCGA database (http://gepia2.cancer-pku.cn/) suggested that the LSP1 gene was highly correlated with the FCGR1A gene, with a Pearson correlation coefficient of 0.76. The expression of the LSP1 gene was significantly greater in OC ovaries compared with normal ovaries, and the difference was statistically significant (see Fig. 4B, C and D, attached Table 3).

## FCGR1A promotes the abdominal metastasis of OC by regulating LSP1 expression

The qRT‒PCR results indicated that the mRNA expression level of LSP1 decreased significantly after FCGR1A silencing, and the difference in LSP1 mRNA expression between the FCGR1A-sh group and the control group was statistically significant (*P* < 0.05). However, the overexpression of LSP1 in OC cells did not result in a concomitant increase in FCGR1A expression. The results of the wound healing and Transwell invasion assays showed that the upregulation of LSP1 significantly promoted cell migration and invasion. The number of migrating OC cells and the number of invading OC cells in the FCGR1A-silenced group were decreased compared with those in the control group, and the inhibitory effect of FCGR1A knockdown on the migration of OC cells could be reversed when LSP1 was overexpressed, which manifested as significant increases in the number of migrating cells and the number of invading cells compared with those in the FCGR1A-silenced group. The results above indicated that LSP1 plays a role in the regulation of OC cell behavior by FCGR1A.

**Fig. 5:**
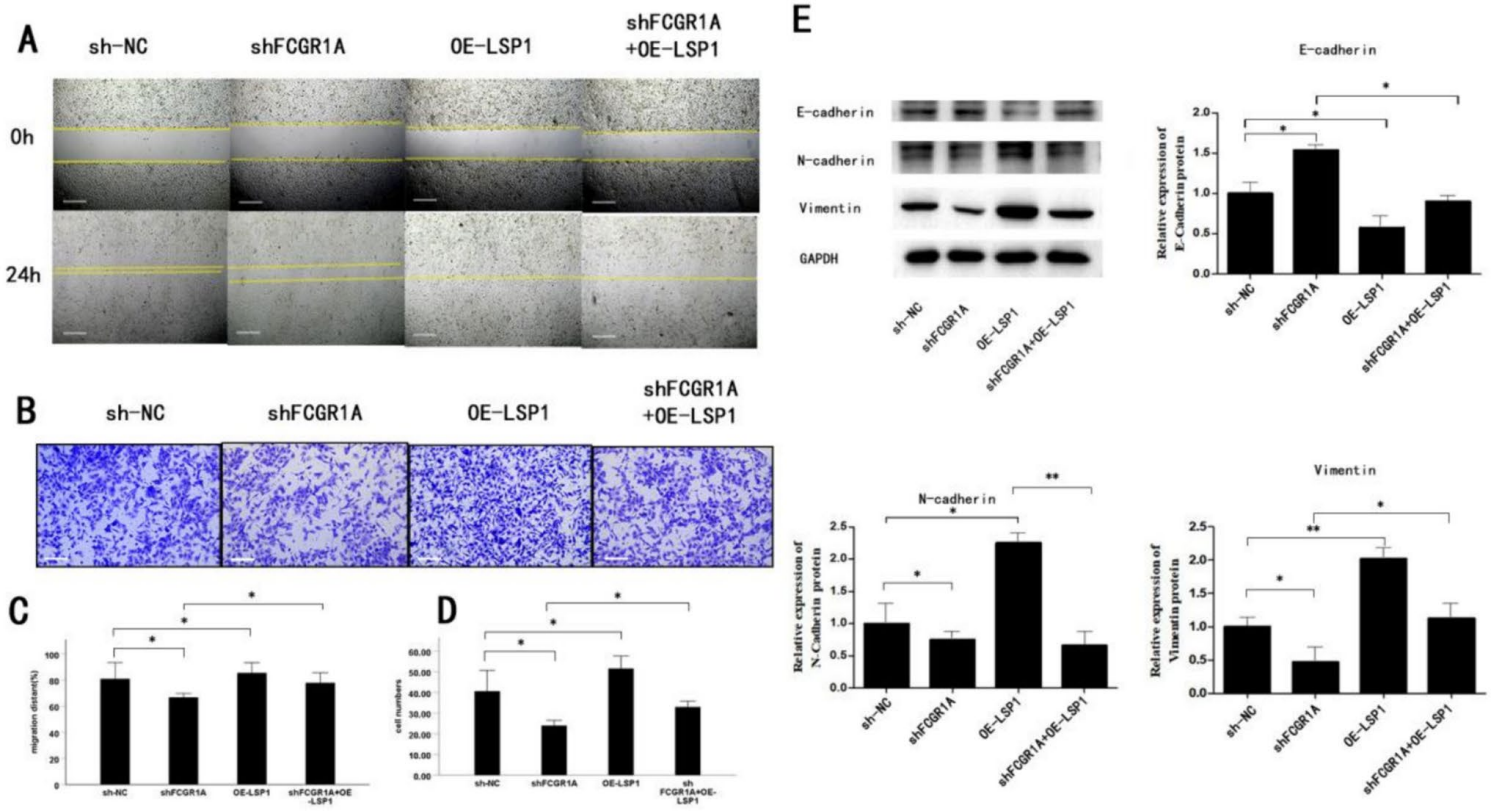
FCGR1A promotes the abdominal metastasis of OC by regulating LSP1 expression. **A–D** Images and quantification of wound healing and Transwell invasion assays in the sh-NC, sh-FCGR1A, LSP-overexpressing, and sh-FCGR1A plus LSP-overexpressing groups. **E** Images and quantification of western blot analysis of E-cadherin, N-cadherin and vimentin in the sh-NC, sh-FCGR1A, LSP-overexpressing, and sh-FCGR1A plus LSP-overexpressing groups (EMT, epithelial-mesenchymal transition. The error bars indicate the SD of the mean from three independent experiments, *P < 0.05, **P < 0.01 by unpaired, two-tailed student's *t*-test. Scale bar = 100 µm)

Furthermore, the upregulation of LSP1 significantly increased the protein expression of N-cadherin and vimentin and decreased the protein expression of E-cadherin compared to that in the control group, and the inhibitory effect on the EMT pathway due to the knockdown of FCGR1A could be reversed when LSP1 was overexpressed. In conclusion, the results suggest that FCGR1A promotes EMT progression in OC cells via LSP1 (see Fig. 5).

## The expression of FCGR1A in OC tissues is correlated with advanced disease stage and worse outcomes.

To evaluate these findings in clinical specimens, a TMA containing 149 epithelial OC tissues was stained with anti-FCGR1A antibody for immunohistochemical analysis. FCGR1A was more highly expressed in metastatic OC patients than in primary OC patients. We further analyzed the relationships between FCGR1A and clinical features and found that the expression of FCGR1A was positively correlated with a higher tumor grade and stage and lymph node metastasis (see Table 1).

**Fig. 6:**
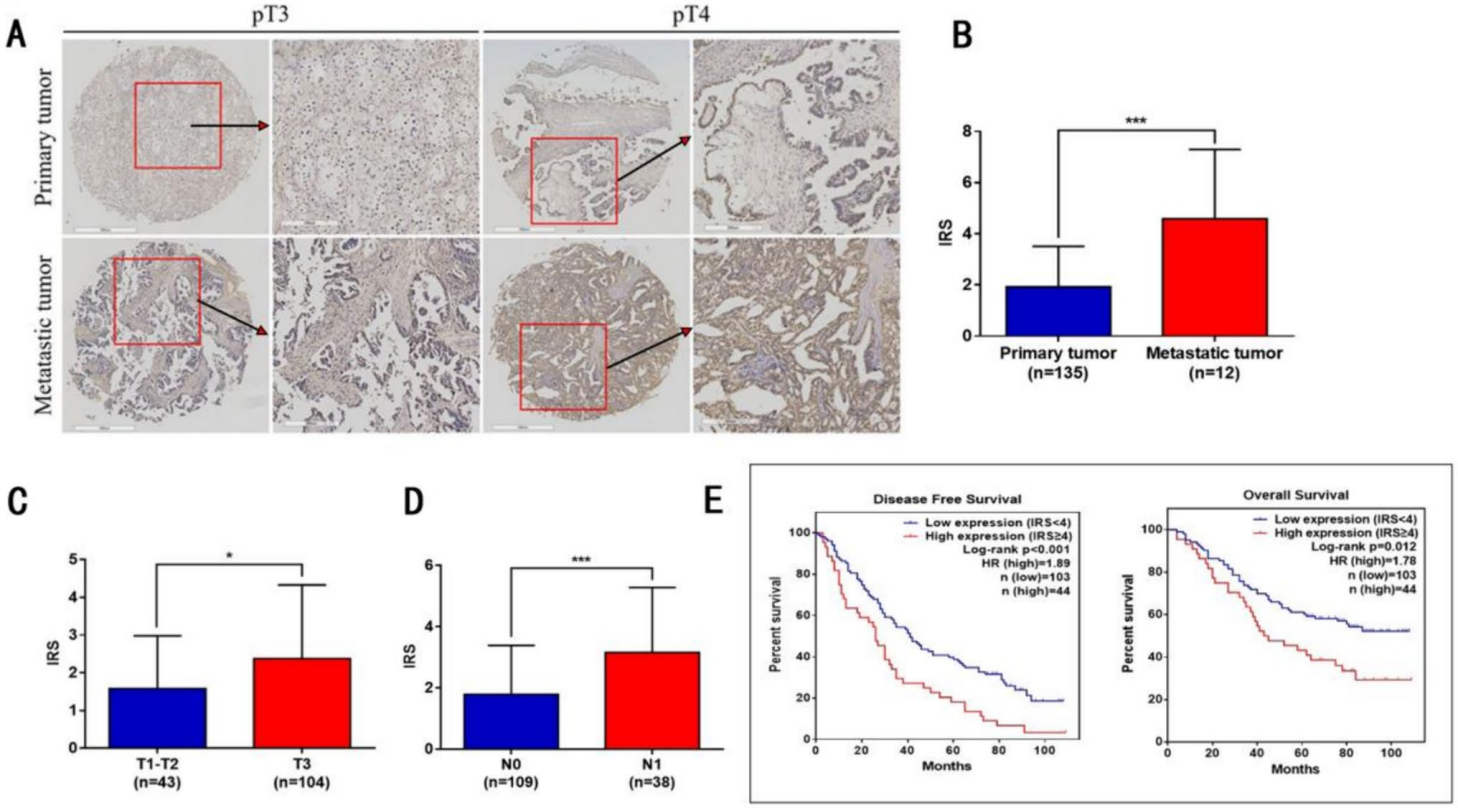
The expression of FCGR1A in OC tissues is correlated with advanced disease stage and worse outcomes. **A** IHC staining showing the expression level of FCGR1A in OC and normal ovarian tissues. **B** IRC scores of FCGR1A in primary and metastatic ovarian tumors. **C** IRC scores of FCGR1A in T1/T2 and T3 ovarian tumors.** D** IRC scores of FCGR1A with different lymph nodes metastasis statuses. **E** Kaplan–Meier analysis of overall survival and disease-free survival in OC patients according to ALKBH5 expression (The error bars indicate the SD of the mean from three independent experiments, *p < 0.05, **p < 0.01 by unpaired, two-tailed Student's t test. Scale bar of the original image in (A) = 500 µm; Scale bar of the magnified image in (A) = 200 µm)

Thereafter, we examined whether the expression levels of FCGR1A were associated with overall survival in OV patients. Kaplan–Meier curves revealed that the group with high FCGR1A expression had a lower tumor-free survival rate (hazard ratio of death from OC, HR 1.886, 95% CI 1.395–3.302, *p* < 0.05) and overall survival rate (HR, 1.776, 95% CI 1.158–3.189, *p* < 0.05) than did the group with low FCGR1A expression. These results indicate that FCGR1A is a potential indicator of poor outcomes (see Fig. 6).

## Discussion

Abdominal metastasis is a major cause of poor prognosis in OC patients (Channawi et al. 2023a). Our study is the first to identify FCGR1A as a promoter of metastasis in ovarian cancer through the regulation of LSP1 genes. In this study, we designed a genome-wide CRISPR/Cas9 screen to identify potential genes that may be involved in OC metastasis. FCGR1A was identified as a key gene that regulates metastasis. We found that FCGR1A enhances cell migration, invasion and proliferation. According to the clinical sample microarray data, FCGR1A was positively correlated with higher tumor grade and stage in OC; moreover, the group with high FCGR1A expression had lower tumor-free survival and overall survival rates than the group with low FCGR1A expression. These results suggested that FCGR1A has the potential to promote the metastasis of OC at the clinical and cellular levels and that FCGR1A may be an indicator of poor outcomes.

Lymph node (LN) metastasis is a common route of metastasis in patients with ovarian cancer and is associated with poor prognosis (Sun et al. 2023). In our present study, the results suggested that the expression level of FCGR1A was greater in metastatic OC than in OC in situ. Notably, the incidence of lymph node metastasis in the high-FCGR1A group was greater than that in the low-FCGR1A group, which suggests that FCGR1A not only facilitates abdominal metastasis but also may facilitate lymph node metastasis through a certain mechanism. Furthermore, the results of the transcriptome analysis suggested that LSP1 is highly correlated with FCGR1A, as silencing FCGR1A resulted in the downregulation of LSP1 expression, while the overexpression of LSP1 could rescue the functional suppression caused by FCGR1A silencing in OC cells. This suggests that FCGR1A promotes tumor invasion by regulating LSP1, making it possible for FCGR1A to promote metastasis by modulating the function of immune cells in the peritoneal microenvironment. In previous studies, LSP1 was reported to be involved in immune escape, and LSP1 secreted by abnormally activated tumor cells regulates metastasis-associated macrophages (Channawi et al. 2023b). The lymphocyte-specific protein LSP1 regulates macrophage phagocytosis and the migration of immune cells. In a study of melanoma, LSP1 in T cells was reported to promote tumor growth by affecting the migration and infiltration capacity of T cells (Galon and Bruni 2019b). LSP1 increased the transcript levels of immunosuppressive genes, such as programmed cell death 1 (pd1) and leukocyte-associated immunoglobulin-like receptor 1 (LAER1), in macrophages (Cao et al. 2020). Maxeiner S et al. investigated the relationship between LSP1 and FCGR1A and showed that LSP1 plays a very important role in FCGR1A-mediated phagocytosis, as the downregulation of LSP1 greatly reduced the phagocytic activity of macrophages (Maxeiner et al. 2015). Therefore, these previous results also support the conclusions of the present study.

Our present study indicated that FCGR1A was related to the EMT progression of OC cells. Type III EMT is associated with tumor progression and metastasis and is a key process in the development of infiltrative metastasis. N-cadherin and vimentin are markers of mesenchymal cells, and their expression suggests that tumor cells have acquired the ability to transform into mesenchymal cells (Yang et al. 2023). According to previous studies, vimentin expression is upregulated during EMT in OC, and blocking vimentin expression leads to the re-epithelialization of cells undergoing EMT and attenuates the invasive ability of tumor cells (Loh et al. 2019). In the present study, the results of a pathway activation assay indicated that FCGR1A is strongly correlated with the EMT process. In vitro assays suggested that both N-cadherin and vimentin were downregulated after silencing FCGR1A, suggesting that FCGR1A promotes the metastasis of OC by regulating the EMT process. In an animal model of abdominal metastasis, the number of metastases was significantly decreased after silencing the FCGR1A gene, which also supported the conclusion that FCGR1A promotes abdominal metastasis in vivo. The inhibitory effect on EMT caused by silencing FCGR1A could be reversed after the overexpression of LSP1 in OC cells. The results above support the hypothesis that FCGR1A promotes EMT progression through its downstream gene LSP1. The above results suggest that FCGR1A may be a molecular indicator for the prediction of multidirectional metastasis in OC.

Although our study provides new insights into the role of FCGR1A in ovarian cancer metastasis, the ability of FCGR1A to promote OC metastasis via LSP1 was verified only at the cellular level and in animal experiments, but the binding site between the two was not further explored; this topic will be explored in depth in subsequent experiments to analyze the mechanism by which metastasis is promoted.

## Conclusion

In summary, this study verified that FCGR1A is associated with advanced-stage lymphatic metastasis and a poor prognosis in patients with ovarian cancer. FCGR1A promotes the metastasis of ovarian cancer cells The mechanism by which FCGR1A promotes abdominal metastasis in ovarian cancer patients is related to the regulation of EMT via LSP1. The detection of FCGR1A is a potential marker for predicting occult abdominal metastases in recurrent ovarian cancer patients.

### Supplementary Information

Below is the link to the electronic supplementary material.

Supplementary file1 (XLSX 38 KB)

Supplementary file2 (DOCX 17 KB)

Supplementary file3 (DOCX 13 KB)

## Data Availability

All data generated or analyzed in this study were included in this published article.

## References

[CR1] Adelmann CH (2019). Genome-Wide CRISPR/Cas9 Screening for Identification of Cancer Genes in Cell Lines. Methods Mol Biol.

[CR2] Bao H (2023). Integrated bioinformatics and machine-learning screening for immune-related genes in diagnosing non-alcoholic fatty liver disease with ischemic stroke and RRS1 pan-cancer analysis. Front Immunol.

[CR3] Cao JY (2020). Elevated lymphocyte specific protein 1 expression is involved in the regulation of leukocyte migration and immunosuppressive microenvironment in glioblastoma. Aging (albany NY).

[CR4] Casamassimi A, Ciccodicola A (2019). Transcriptional Regulation: Molecules, Involved Mechanisms, and Misregulation. Int J Mol Sci.

[CR5] Channawi A (2023). Prognostic Impact of Mesenteric Lymph Node Status on Digestive Resection Specimens During Cytoreductive Surgery for Ovarian Peritoneal Metastases. Ann Surg Oncol.

[CR6] Channawi A (2023). Prognostic Impact of Mesenteric Lymph Node Status on Digestive Resection Specimens During Cytoreductive Surgery for Ovarian Peritoneal Metastases. Ann Surg Oncol.

[CR7] Curnow RT (1997). Clinical experience with CD64-directed immunotherapy An Overview. Cancer Immunol Immunother.

[CR8] DiSilvestro P (2023). Overall Survival With Maintenance Olaparib at a 7-Year Follow-Up in Patients With Newly Diagnosed Advanced Ovarian Cancer and a BRCA Mutation: The SOLO1/GOG 3004 Trial. J Clin Oncol.

[CR9] Galon J, Bruni D (2019). Approaches to treat immune hot, altered and cold tumours with combination immunotherapies. Nat Rev Drug Discov.

[CR10] Galon J, Bruni D (2019). Approaches to treat immune hot, altered and cold tumours with combination immunotherapies. Nat Rev Drug Discov.

[CR11] Gorzelnik K (2023). Expression of B7–H4 in endometrial cancer and its impact on patients' prognosis. Ginekol Pol.

[CR12] Jia X (2021). CD47/SIRPα pathway mediates cancer immune escape and immunotherapy. Int J Biol Sci.

[CR13] Kaushal P (2023). Prognosis and Immune Landscapes in Glioblastoma Based on Gene-Signature Related to Reactive-Oxygen-Species. Neuromolecular Med.

[CR14] Loh CY (2019). The E-Cadherin and N-Cadherin Switch in Epithelial-to-Mesenchymal Transition: Signaling, Therapeutic Implications, and Challenges. Cells.

[CR15] Maxeiner S, Shi N, Schalla C (2015). Crucial role for the LSP1-myosin1e bimolecular complex in the regulation of Fcgamma receptor-driven phagocytosis. Mol Biol Cell.

[CR16] Pennington K (2013). Germline and Somatic Mutations in Homologous Recombination Genes Predict Platinum Response and Survival in Ovarian, Fallopian Tube, and Peritoneal Carcinomas. Clin Cancer Res.

[CR17] Repp R (1995). G-CSF-stimulated PMN in immunotherapy of breast cancer with a bispecific antibody to Fc gamma RI and to HER-2/neu (MDX-210). J Hematother.

[CR18] Ronsini C (2023). Minimally Invasive Staging of Early-Stage Epithelial Ovarian Cancer versus Open Surgery in Terms of Feasibility and Safety: A Systematic Review and Meta-Analysis. J Clin Med.

[CR19] Scartozzi M (2010). Dalotuzumab, a recombinant humanized mAb targeted against IGFR1 for the treatment of cancer. Curr Opin Mol Ther.

[CR20] Sun (2023). ALKBH5 activates FAK signaling through m6A demethylation in ITGB1 mRNA and enhances tumor-associated lymphangiogenesis and lymph node metastasis in ovarian cancer. Theranostics.

[CR21] Yang X (2023). Histone acetyltransferase CSRP2BP promotes the epithelial-mesenchymal transition and metastasis of cervical cancer cells by activating N-cadherin. J Exp Clin Cancer Res.

[CR22] Zhang H (2018). FcγRI (CD64) contributes to the severity of immune inflammation through regulating NF-κB/NLRP3 inflammasome pathway. Life Sci.

