# Supplementary material for: CRISPR/Cas9-based genome-wide screening for metastasis ability identifies FCGR1A regulating the metastatic process of ovarian cancer by targeting LSP1
Source: J Cancer Res Clin Oncol. 2024 Jun 15;150(6):306. doi: 10.1007/s00432-024-05837-9 (PMC11180010; doi:10.1007/s00432-024-05837-9)
Supplement: Supplementary file 2 — Supplementary file2 (DOCX 17 KB) [file 432_2024_5837_MOESM2_ESM.docx]

**Attached table 2 The Top 20 Upregulated and** **Downregulated Genes**

| **Upregulated Genes** | **log2(fc)** | **Function** | **P-Value** | **Rank** |
| --- | --- | --- | --- | --- |
| MEPCE | 7.037089319 | Up | 0.002964455 | 1 |
| ZNF799 | 3.676705069 | Up | 0.008422849 | 2 |
| ANGPT2 | 1.957163147 | Up | 2.49E-41 | 3 |
| NDUFA7 | 1.720584453 | Up | 0.00405374 | 4 |
| PPP1R1A | 1.532534808 | Up | 0.00373945 | 5 |
| POLA2 | 1.387336904 | Up | 0.00295974 | 6 |
| LY75-CD302 | 1.364296734 | Up | 0.00013418 | 7 |
| BGN | 1.350055477 | Up | 6.16E-07 | 8 |
| SULT1E1 | 1.331487431 | Up | 6.84E-05 | 9 |
| SHISA3 | 1.177068431 | Up | 6.50E-05 | 10 |
| FAM24B | 1.169966114 | Up | 0.009784074 | 11 |
| SLC14A1 | 1.035759763 | Up | 0.002122944 | 12 |
| ESM1 | 1.033981 | Up | 1.33E-05 | 13 |
| HSD17B10 | 1.008690379 | Up | 2.85E-17 | 14 |
| LUM | 0.985725087 | Up | 1.05E-06 | 15 |
| CCL26 | 0.934127679 | Up | 0.007664587 | 16 |
| THBD | 0.906736522 | Up | 1.33E-15 | 17 |
| TNC | 0.900312103 | Up | 1.36E-07 | 18 |
| AMTN | 0.892708563 | Up | 1.41E-36 | 19 |
| EVI5L | 0.883193259 | Up | 4.14E-08 | 20 |

| **Downregulated Genes** | **log2(fc)** | **Function** | **P-Value** | **Rank** |
| --- | --- | --- | --- | --- |
| MAGEA12 | -7.684164178 | down | 0.003108389 | 1 |
| CXCL6 | -7.594946589 | down | 0.006732514 | 2 |
| IRF6 | -4.765775818 | down | 4.87E-08 | 3 |
| LSP1 | -2.263034406 | down | 0.001100865 | 4 |
| TMEFF2 | -2.031922405 | down | 0.005819551 | 5 |
| OLFML2B | -1.882960579 | down | 0.002108228 | 6 |
| TNNT2 | -1.762298906 | down | 0.003975076 | 7 |
| CDHR1 | -1.671335102 | down | 1.09E-05 | 8 |
| QRFPR | -1.657418567 | down | 0.000766109 | 9 |
| -- | -1.650845062 | down | 0.000192808 | 10 |
| LRG1 | -1.594082689 | down | 3.00E-06 | 11 |
| C2orf88 | -1.446552054 | down | 2.98E-19 | 12 |
| ALDH1A1 | -1.408430771 | down | 0.003633492 | 13 |
| TIE1 | -1.389042291 | down | 0.002468394 | 14 |
| KCNE3 | -1.386609091 | down | 2.14E-06 | 15 |
| MUC16 | -1.377288223 | down | 1.65E-05 | 16 |
| CLDN2 | -1.350359442 | down | 0.00037118 | 17 |
| COL9A2 | -1.340366498 | down | 7.78E-07 | 18 |
| LYNX1 | -1.316857105 | down | 3.53E-05 | 19 |
| DOK7 | -1.244186411 | down | 4.83E-05 | 20 |
