# Supplementary material for: CRISPR/Cas9-based genome-wide screening for metastasis ability identifies FCGR1A regulating the metastatic process of ovarian cancer by targeting LSP1
Source: J Cancer Res Clin Oncol. 2024 Jun 15;150(6):306. doi: 10.1007/s00432-024-05837-9 (PMC11180010; doi:10.1007/s00432-024-05837-9)
Supplement: Supplementary file 3 — Supplementary file3 (DOCX 13 KB) [file 432_2024_5837_MOESM3_ESM.docx]

**Attached table 4 Pearson related analysis of FCGR1A and up/down regulated genes**

| **Up** | **Co-expreession（Pearson）** | **Down** | **Co-expreession（Pearson）** |
| --- | --- | --- | --- |
| MEPCE | -0.089 | MAGEA12 | −0.07 |
| ZNF799 | −0.064 | CXCL6 | 0.14 |
| ANGPT2 | 0.04 | IRF6 | 0.097 |
| NDUFA7 | −0.091 | LSP1 | 0.7 |
| PPP1R1A | −0.13 | TMEFF2 | −0.038 |
| POLA2 | 0.064 | OLFML2B | 0.43 |
| LY75-CD302 | 0.045 | TNNT2 | −0.052 |
| BGN | 0.29 | CDHR1 | −0.017 |
| SULT1E1 | −0.035 | QRFPR | −0.064 |
| SHISA3 | −0.014 | CHD9NB | 0.037 |
| FAM24B | −0.16 | LRG1 | 0.22 |
| SLC14A1 | −0.04 | C2orf88 | 0.032 |
| ESM1 | 0.048 | ALDH1A1 | −0.34 |
| HSD17B10 | 0.079 | TIE1 | −0.093 |
| LUM | 0.18 | KCNE3 | 0.029 |
| CCL26 | −0.26 | MUC16 | 0.18 |
| THBD | 0.093 | CLDN2 | −0.056 |
| TNC | 0.16 | COL9A2 | −0.07 |
| AMTN | 0.18 | LYNX1 | 0.075 |
| EVI5L | 0.01 | DOK7 | 0.15 |
